# FIZZ1 and Ym as Tools to Discriminate between Differentially Activated Macrophages

**DOI:** 10.1080/1044667031000137629

**Published:** 2002-09

**Authors:** Geert Raes, Wim Noël, Alain Beschin, Lea Brys, Patrick de Baetselier, Gh. Gholamreza Hassanzadeh

**Affiliations:** Department of Molecular and Cellular Interactions Vlaams Interuniversitair Instituut voor Biotechnologie Vrije Universiteit Brussel Pleinlaan 2 Brussels B-1050 Belgium

## Abstract

Although it is well-established that macrophages can occur in distinct activation states, the molecular characteristics of differentially activated macrophages, and particularly those of alternatively activated macrophages (aaMφ), are still poorly unraveled. Recently, we demonstrated that the expression of FIZZ1 and Ym is induced in aaMφ as compared with classically activated macrophages (caMφ), elicited *in vitro* or developed *in vivo* during infection with *Trypanosoma brucei brucei*. In the present study, we analyzed the expression of FIZZ1 and Ym in caMφ and aaMφ elicited during *Trypanosoma congolense* infection and show that the use of FIZZ1 and Ym for the identification of aaMφ is not limited to *T. b. brucei* infection and is independent of the organ sources from which macrophages are obtained. We also demonstrate that FIZZ1 can be used to discriminate between different populations of aaMφ. Furthermore, we studied the effects of various stimuli, and combinations thereof, on the expression of FIZZ1 and Ym in macrophages from different mouse strains and demonstrate that regulation of the expression of FIZZ1 and Ym in macrophages is not dependent on the mouse strain. Finally, we show that these genes can be used to monitor the macrophage activation status without the need to obtain pure macrophage populations.


*Developmental Immunology*, September 2002, Vol. 9 (3), pp. 151–159

Taylor & Francis

Taylor & Francis Group

### FIZZ1 and Ym as Tools to Discriminate between Differentially Activated Macrophages*

GEERT RAES, WIM NOËL, ALAIN BESCHIN, LEA BRYS, PATRICK DE BAETSELIER and GH. GHOLAMREZA HASSANZADEH†

*Department of Molecular and Cellular Interactions, Vlaams Interuniversitair Instituut voor Biotechnologie, Vrije Universiteit Brussel, Pleinlaan 2, B-1050 Brussels, Belgium*

Although it is well-established that macrophages can occur in distinct activation states, the molecular characteristics of differentially activated macrophages, and particularly those of alternatively activated macrophages (aaMϕ), are still poorly unraveled. Recently, we demonstrated that the expression of FIZZ1 and Ym is induced in aaMϕ as compared with classically activated macrophages (caMϕ), elicited *in vitro* or developed *in vivo* during infection with *Trypanosoma brucei brucei*. In the present study, we analyzed the expression of FIZZ1 and Ym in caMϕ and aaMϕ elicited during *Trypanosoma congolense* infection and show that the use of FIZZ1 and Ym for the identification of aaMϕ is not limited to *T. b. brucei* infection and is independent of the organ sources from which macrophages are obtained. We also demonstrate that FIZZ1 can be used to discriminate between different populations of aaMϕ. Furthermore, we studied the effects of various stimuli, and combinations thereof, on the expression of FIZZ1 and Ym in macrophages from different mouse strains and demonstrate that regulation of the expression of FIZZ1 and Ym in macrophages is not dependent on the mouse strain. Finally, we show that these genes can be used to monitor the macrophage activation status without the need to obtain pure macrophage populations.

*Keywords*: Gene expression; Macrophage activation; *Trypanosoma*; L-Arginine

### INTRODUCTION

As for many other cell types of the immune system, macrophages can exist under the form of different subsets exhibiting different functional and molecular properties. The emergence of different macrophage subsets is primarily due to differences in the environments to which macrophages are exposed (Henson and Riches, 1994; Erwig *et al.*, 1998; for review, see Goerdt and Orfanos, 1999). The best-studied macrophage subsets are classically activated macrophages (caMϕ) that differentiate in the presence of proinflammatory stimuli such as type I cytokines and LPS (Goerdt and Orfanos, 1999). Classically activated macrophages possess anti-microbial, anti-proliferative and cytotoxic properties, partly due to their ability to secrete nitric oxide (NO) and proinflammatory cytokines such as TNF, IL-1 and IL-6 (Janeway and Travers, 1996). Although caMϕ are important components of host defense in the fight against various pathogens, the persistence or escalation of the inflammatory processes mediated by caMϕ can result in immunopathology (Gazzinelli *et al.*, 1996; Li *et al.*, 1999). Thus, for an immune response to be as beneficial to the host as possible, the resolution of inflammation is of crucial importance (Hunter *et al.*, 1997; Namangala *et al.*, 2000; De Baetselier *et al.*, 2001). Type II immune response mediators, such as IL-4 and glucocorticoids, antagonize classical macrophage activation and induce the development of alternatively activated macrophages (aaMϕ) (Goerdt and Orfanos, 1999). In aaMϕ, inducible nitric oxide synthase (iNOS), catalyzing the production of NO and L-citrulline from L-arginine, is suppressed; instead, in an alternative pathway, catalyzed by arginase, L-arginine is converted to L-ornithine and urea (Munder *et al.*, 1998). Moreover, alternatively activated macrophages secrete anti-inflammatory mediators such as TGF-β and IL-10 (Goerdt and Orfanos, 1999). On this basis, aaMϕ have been considered to secure the balance between pro- and anti-inflammatory reactions during type I cytokine-driven inflammatory responses (Goerdt and Orfanos, 1999). In addition, it has been reported that aaMϕ exhibit a high endo- and phagocytotic capacity (Goerdt and Orfanos, 1999), can promote angiogenesis (Kodelja *et al.*, 1997) and contribute to wound healing (Gratchev *et al.*, 2001). However, the exact functional properties of aaMϕ *in vivo* remain unclear. Indeed, aaMϕ have been found to be associated

*Presented at the Proceedings of the 4th Germinal Center Conference, Groningen, The Netherlands, June 2002.
†Corresponding author. Tel.: +32-2-629-19-80. Fax: +32-2-629-19-81. E-mail: reza@ben.vub.ac.be

ISSN 1044-6672 print © 2002 Taylor & Francis Ltd
DOI: 10.1080/1044667031000137629

with type II cytokine-controlled inflammatory diseases (Loke *et al.*, 2000a; Falcone *et al.*, 2001). Therefore, it cannot be excluded that, under these circumstances, aaMϕ support the development of pathology. Finally, because of their ability to act as antigen presenting cells as well as to secrete immunosuppressive and/or immunomodulatory mediators, caMϕ and aaMϕ can influence the development and maintenance of adaptive immune responses (Janeway and Travers, 1996; Schebesch *et al.*, 1997; Namangala *et al.*, 2000; Loke *et al.*, 2000b).

The molecular differences between differentially activated macrophages have slowly begun to be unraveled. For example, expression of the three species of Fcγ receptor on macrophages was found to be induced by IFN-γ but inhibited by IL-4 (Becker and Daniel, 1990; Te Velde *et al.*, 1990). On the other hand, the macrophage mannose receptor (Stein *et al.*, 1992), 15-lipoxygenase (Conrad *et al.*, 1992), fibronectin and the extracellular matrix protein βIG-H3 (Gratchev *et al.*, 2001) have been shown to be up-regulated in IL-4-induced aaMϕ. In human macrophages, the surface markers MS-1 high molecular weight protein (MS-1-HMWP) (Goerdt *et al.*, 1993), the scavenger receptor CD163 (RM3/1 antigen) (Buechler *et al.*, 2000) and the chemokine AMAC-1 (Kodelja *et al.*, 1998) are thus far the most commonly used markers to characterize the alternative pathway of activation. Molecular characterization of aaMϕ and caMϕ has so far been mainly carried out using differentially activated macrophages obtained by *in vitro* steering of human peripheral blood monocytes. However, the *in vivo* environments in which macrophages develop are far more complex than the environments generated *in vitro*. Hence, the differential gene expression data obtained from *in vivo*-elicited aaMϕ and caMϕ may be more physiologically relevant than those obtained from *in vitro*-induced macrophages. *In vivo* murine models can be used to study the mechanisms underlying the functional properties of different macrophage populations and their differential activation, in ways that would not be conceivable in humans. Further characterization of the molecular repertoire of differentially activated macrophages is a prerequisite for a better understanding of the mode of action and differential activation of macrophages, which in turn may open new avenues towards the development of novel diagnostic and therapeutic approaches. However, in mouse, molecular markers for the identification of different macrophage populations are scarce and discrimination between murine caMϕ and aaMϕ has so far been based mainly on differential arginine metabolism via iNOS and arginase (Munder *et al.*, 1998). Recently, we focused on the identification of genes that are differentially expressed in aaMϕ versus caMϕ elicited during murine PLC$^{-/-}$ *Trypanosoma brucei brucei* infection (Raes *et al.*, 2002). In this infection model, correlating with a switch from a type I cytokine environment in the early stage of infection to a type II cytokine environment in the late and chronic phases, macrophages from early stage-infected mice are caMϕ, and those from the late and chronic stages of infection are aaMϕ (De Baetselier *et al.*, 2001; Namangala *et al.*, 2001). Using this infection model, we demonstrated that the expression of FIZZ1, also referred to as RELMα, (Holcomb *et al.*, 2000; Steppan *et al.*, 2000) and Ym (Guo *et al.*, 2000; Webb *et al.*, 2001; Chang *et al.*, 2001a) is induced strongly in *in vivo*-elicited adherent peritoneal and splenic aaMϕ as compared to caMϕ (Raes *et al.*, 2002).

In the present study, we analyzed the expression patterns of FIZZ1 and Ym in different macrophage populations elicited during *Trypanosoma congolense* infection. In contrast to *T. b. brucei*, that is a tissue-infiltrating parasite, *T. congolense* remains mainly in the vasculature (Black and Seed, 2001). Moreover, whereas essentially the same course of infection is recorded upon inoculation of *T. b. brucei* in C57Bl/6 or BALB/c mice, there is a clear difference in susceptibility of these two mouse strains towards infection with *T. congolense* (Uzonno *et al.*, 1999). Hence, in this study, we evaluated FIZZ1 and Ym as markers for macrophage activation status in models for resistance versus susceptibility towards parasite infection. Furthermore, we tested the effects of type I and type II stimuli on the expression of FIZZ1 and Ym in macrophages derived from different mouse strains.

### RESULTS

### Differential Expression of FIZZ1 and Ym in Macrophages Elicited During *T. congolense* Infection

In a model of *T. congolense* infection, BALB/c mice are considered as extremely susceptible, dying within 8 ± 2 days of infection, following uncontrolled exponential parasite growth (Uzonna *et al.*, 1999). Spleen cells from early-stage (6 days post-infection) *T. congolense*-infected BALB/c mice secreted high levels of IFN-γ, IL-4 and IL-13 (Fig. 1A). Splenic macrophages developed in this mixed type I/type II cytokine environment exhibited high levels of both arginase activity and NO secretion, reflecting a mixed classically/alternatively activated phenotype (Fig. 1B, C). As compared with splenic macrophages from non-infected BALB/c mice, Ym, but not FIZZ1, was strongly induced in splenic macrophages from early stage-infected BALB/c animals (Fig. 1D).

In contrast to BALB/c mice, C57Bl/6 mice are resistant to *T. congolense* infection, exhibiting low parasitemia and a survival time of 160 ± 25 days (Uzonno *et al.*, 1999). In *T. congolense*-infected C57Bl/6 mice, the level of IFN-γ produced by spleen cells was higher in the early stage (6 days post-infection) of infection than in the late stage (8 weeks post-infection) while IL-4 and IL-13 secretions were higher in the late than in the early stage (Fig. 1A). Therefore, spleen cells from early- and late-stage *T. congolense*-infected C57Bl/6 mice exhibit predominant type I and predominant type II cytokine profiles, respectively. Accordingly, based on arginase activity

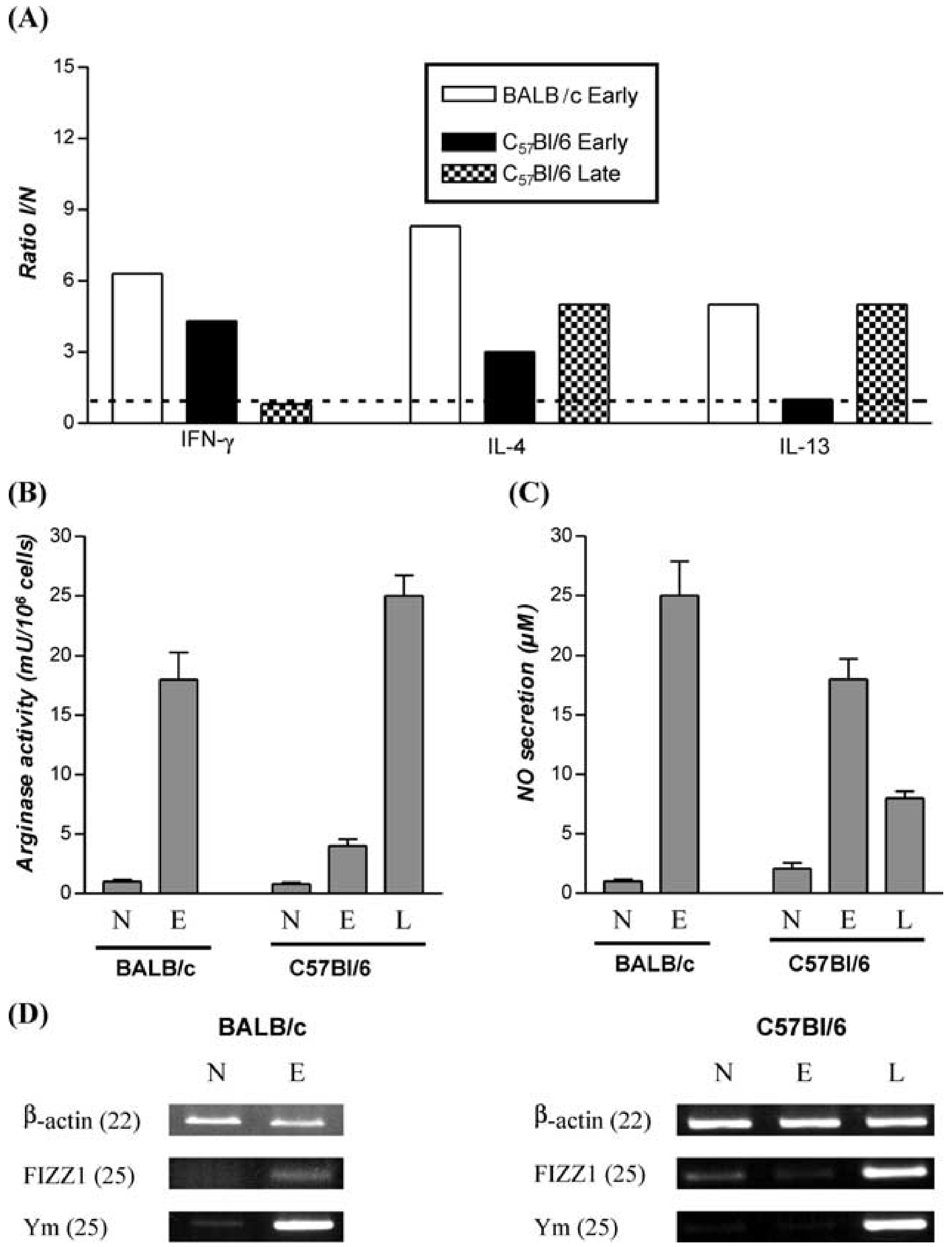

FIGURE 1 Cytokine profiles, macrophage activation status and FIZZ1/Ym expression in the spleens of *T. congolense*-infected mice. (A) Cytokine profiles of the spleens from early-stage *T. congolense*-infected BALB/c mice and early- and late-stage *T. congolense*-infected C57Bl/6 mice. The data are presented as the ratio of cytokine secretion between spleen cells from infected (I) and non-infected (N) mice. The dotted line indicates an I/N ratio of 1. (B) Arginase activity and (C) nitric oxide (NO) secretion levels in splenic macrophages from non-infected (N), early stage (E) and late phase (L) *T. congolense*-infected animals. (D) Semi-quantitative RT-PCR analysis of FIZZ1 and Ym expression in adherent splenocytes from non-infected (lane N), early (lane E) and late (lane L) stage *T. congolense*-infected C57BL/6 or BALB/c mice. The number of PCR cycles, indicated in parenthesis, was optimized so that PCR analysis could be carried out within the linear phase of amplification. The house-keeping gene β-actin was used to compare the amount of cDNA templates used.

and NO secretion levels, splenic macrophages from the early and late stages of infection of these C57Bl/6 mice can be classified as caMϕ and aaMϕ, respectively (Fig. 1B, C). As compared with macrophages from non-infected C57Bl/6 animals and caMϕ developed in the early stage of *T. congolense* infection, the expression of FIZZ1 and Ym was strongly induced in splenic aaMϕ elicited in the late stage of infection (Fig. 1D).

### Regulation of the Expression of FIZZ1 and Ym in Macrophages is Independent of Mouse Strain

Results in the preceding section demonstrate that, during *T. congolense* infection, the expression of FIZZ1 was induced in macrophages developed during the late stage of infection in C57Bl/6 mice, but not in macrophages elicited during the early stage of infection in BALB/c mice, despite the fact that both macrophage populations exhibited high arginase activity and expressed high levels of Ym. To test whether the difference in the expression of FIZZ1 in these aaMϕ populations is due to differences in the regulation of this gene in the two mouse strains analyzed, we investigated the effects of various stimuli on the expression of FIZZ1 in macrophages isolated from BALB/c and C57Bl/6 mice.

Adherent PEC from non-infected BALB/c and C57Bl/6 mice elicited with thioglycollate were steered *in vitro* and their activation status was determined by measuring the arginase activity and NO production (Fig. 2A, B). As compared to non-stimulated cells, in both mouse strains, stimulation with IL-4, IL-13, or IL-4 plus IL-13 resulted in enhancement of arginase activity but had no effect on NO secretion. In C57Bl/6, but not in BALB/c mice, IL-10 augmented arginase activity of macrophages, although to a lesser extent than IL-4 or IL-13. IL-10 had no effect on IL-4-induced arginase activity in both mouse strains. IL-10 induction of arginase activity, observed in macrophages from C57Bl/6 mice, was not affected by the simultaneous addition of IFN-γ. In contrast, IFN-γ completely inhibited IL-4 induction of arginase activity in both strains. Simultaneous addition of LPS partially inhibited IL-4-induced arginase activity in BALB/c mice but had no such effect in C57Bl/6 mice (Fig. 2A). Hence, in both BALB/c and C57Bl/6 mice, IL-4 induction of arginase activity was more efficiently inhibited by simultaneous addition of IFN-γ than LPS. As shown in Fig. 2B, in C57Bl/6, as well as in BALB/c mice, stimulation with LPS, IFN-γ, or a combination thereof, resulted in increase in NO secretion, but did not affect the arginase activity. In both strains, IL-4 partially diminished LPS-, but not IFN-γ-induced, NO secretion, whereas IL-10 nearly completely inhibited induction of NO by IFN-γ.

RT-PCR analysis revealed that, as compared with non-stimulated macrophages, stimulation with IL-4, IL-13, IL-4 plus IL-13, or IL-4 plus IL-10 resulted in the induction of FIZZ1 and Ym in macrophages from both BALB/c and C57Bl/6 mice (Fig. 2C, D). In macrophages from both mouse strains, the type I stimulus IFN-γ antagonized completely the effect of IL-4 on the expression of both FIZZ1 and Ym. LPS completely inhibited IL-4-induced FIZZ1, but only partially suppressed IL-4 induction of Ym in both strains (Fig. 2C, D).

Collectively, the above data showed that the *in vitro* steering conditions inducing high expression levels of both FIZZ1 and Ym, resulted in high arginase activity and low NO secretion levels. The expression of Ym, but not FIZZ1, was also elevated in macrophages treated with IL-4 plus LPS, exhibiting increased levels of both arginase activity and NO secretion. The above experiments also indicated that, at the concentrations of modulators that were tested, macrophages derived from C57Bl/6 and BALB/c mice do not differ in the regulation of FIZZ1 and Ym expression upon *in vitro* activation by the various immune modulators.

### FIZZ1 and Ym as Tools to Monitor the Macrophage Activation Status using Total PEC

As compared with caMϕ, both *in vitro*- and *in vivo*-elicited aaMϕ, purified by adherence, express high levels of FIZZ1 and Ym. To test whether these genes can be used to characterize the macrophage activation state without the need to enrich the cell populations for macrophages, we used total PEC from non-infected, early- and chronic-stage $PLC^{-/-}$ *T. b. brucei*-infected F1 mice. As shown in Fig. 3A, B, peritoneal cells from the early stage of infection exhibited low arginase activity and high level of NO secretion, reflecting the presence of caMϕ. Peritoneal cells from the chronic stage of infection secreted marginal levels of NO and displayed high arginase activity, reflecting the presence of aaMϕ. Semi-quantitative RT-PCR analysis revealed that, as compared to total PEC from non-infected and early-stage $PLC^{-/-}$ *T. b. brucei*-infected mice, the expression of FIZZ1 and Ym in total PEC from chronic-stage $PLC^{-/-}$ *T. b. brucei*-infected animals was notably high (Fig. 3C).

### DISCUSSION

FIZZ1 (found in inflammatory zone) is a resistin-like secreted protein which is markedly up-regulated in the lung during allergic pulmonary inflammation (Holcomb *et al.*, 2000; Steppan *et al.*, 2000). Ym is a chitinase-like secretory lectin that forms crystals in the lungs of mice exhibiting hyperactivity of alveolar macrophages (Guo *et al.*, 2000; Sun *et al.*, 2001; Chang *et al.*, 2001a) and is up-regulated in the lung during the development of allergy (Webb *et al.*, 2001). Recently, we demonstrated that the expression of FIZZ1 and Ym genes is induced in aaMϕ developed during $PLC^{-/-}$ *T. b. brucei* infection (Raes *et al.*, 2002). In the present study, we focused on the analysis of the expression of these genes in macrophages

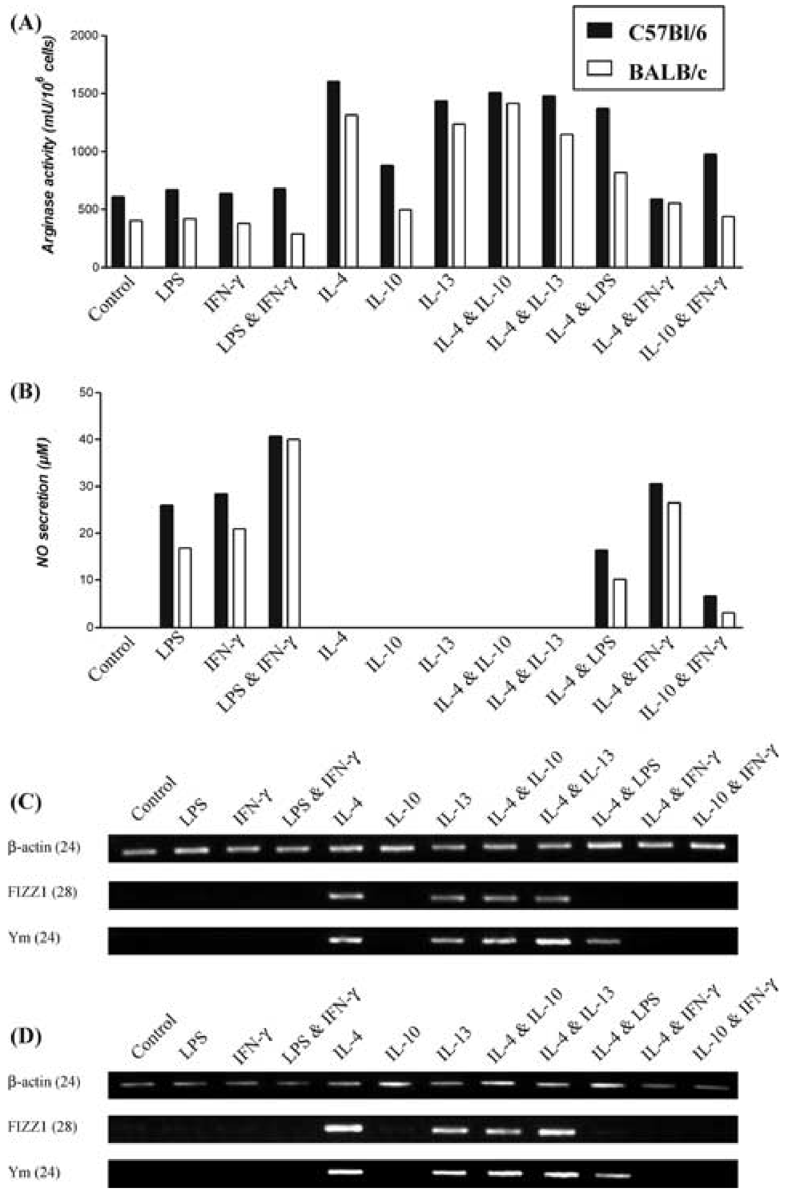

FIGURE 2 Activation status of *in vitro*-elicited differentially activated macrophages. Adherent thioglycollate-elicited peritoneal macrophages from BALB/c and C57Bl/6 mice were cultured without any stimuli (control) or in the presence of the indicated stimuli. (A) Arginase activity. (B) Nitric oxide (NO) secretion levels. (C and D) Semi-quantitative RT-PCR analysis of the expression of FIZZ1 and Ym in macrophages from BALB/c (C) or C57Bl/6 (D) mice. The number of PCR cycles, indicated in parenthesis, was optimized so that PCR analysis could be carried out within the linear phase of amplification. The house-keeping gene β-actin was used to compare the amount of cDNA templates used.

elicited during *T. congolense* infection. It should be remarked that, so far, two different isotypes of Ym, termed Ym1 and Ym2, have been characterized (Guo *et al.*, 2000; Chang *et al.*, 2001a; Sun *et al.*, 2001; Webb *et al.*, 2001). The primers used in our studies amplify both Ym1 and Ym2 sequences. Therefore, our experiments address the expression of Ym in general and not of its particular isotypes.

We showed that, during infection of C57Bl/6 mice with *T. congolense*, correlating with a switch from a predominant type I cytokine profile in the early stage of infection to a predominant type II cytokine environment in the late phase, splenic macrophages from the early stage are caMϕ, and those from the late phase are aaMϕ. As is the case with aaMϕ elicited during PLC$^{-/-}$ *T. b. brucei* infection (Raes *et al.*, 2002), splenic aaMϕ induced during the late stage of infection of C57Bl/6 mice with *T. congolense* exhibit notably high levels of FIZZ1 and Ym expression as compared with macrophages from non-infected and early stage-infected animals. This shows that the use of FIZZ1 and Ym to identify aaMϕ is not limited to PLC$^{-/-}$ *T. b. brucei* infection. Moreover, the enhanced expression of FIZZ1 and Ym in both peritoneal (Noël *et al.*, 2002) and splenic macrophages (documented in this paper) elicited during infection of C57Bl/6 mice with *T. congolense* further confirms our previous findings in the PLC$^{-/-}$ *T. b. brucei* model (Raes *et al.*, 2002) that the expression of these genes is

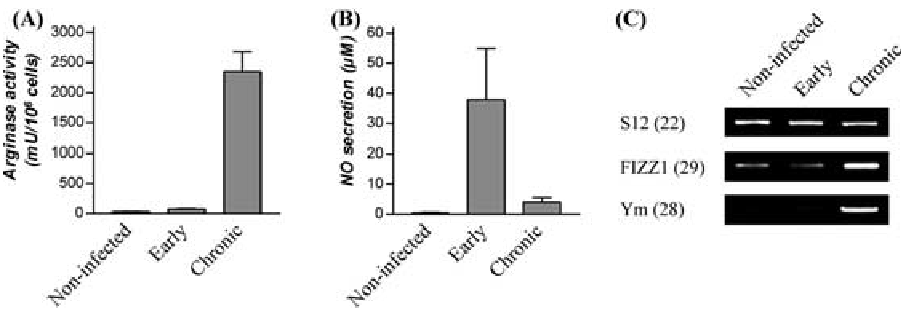

FIGURE 3 Macrophage activation status during infection with PLC$^{-/-}$ *T. b. brucei*. (A) Arginase activity and (B) nitric oxide (NO) secretion in total PEC from non-infected, early-stage and chronic-stage infected F1 mice. (C) Semi-quantitative RT-PCR analysis of FIZZ1 and Ym expression in total PEC from non-infected, early-stage and chronic-stage infected mice. The number of PCR cycles, indicated in parenthesis, was optimized so that PCR analysis could be carried out within the linear phase of amplification. The house-keeping gene ribosomal protein S12 was used to compare the amount of cDNA templates used.

independent of the organ sources from which macrophages are obtained.

Spleens of early-stage *T. congolense*-infected BALB/c mice exhibited a mixed type I/type II cytokine profile and harbored macrophage populations which were similar to aaMϕ from late-stage *T. congolense*-infected C57Bl/6 mice in that their arginase activity and Ym expression level were high. On the other hand, FIZZ1 was induced strongly in aaMϕ from late-stage *T. congolense*-infected C57Bl/6 mice but not in macrophages from early-stage *T. congolense*-infected BALB/c mice. Therefore, FIZZ1 may discriminate between different populations of aaMϕ. Interestingly, in these BALB/c and C57Bl/6 models of *T. congolense* infection, a strong induction of FIZZ1 is associated with resistance. Although, *in vitro*, IFNγ blocked the IL-4-induced expression of arginase, Ym and FIZZ1, the induction of arginase activity and Ym expression, but not FIZZ1 expression, in macrophages from early-stage *T. congolense*-infected BALB/c mice, suggests that IFN-γ suppresses more efficiently the expression of FIZZ1 than YM and arginase in these *in vivo*-elicited aaMϕ.

In general, analysis of *in vitro*-stimulated macrophages revealed that, in regard to FIZZ1 and Ym expression, macrophages derived from C57Bl/6 and BALB/c mice do not differ in their responses to the same stimuli. Thus, the difference in the expression of FIZZ1 between macrophages from early-stage *T. congolense*-infected BALB/c mice and macrophages from late-stage *T. congolense* C57Bl/6 mice is probably due to the differences in the cytokine environments in which macrophages develop. Indeed, in *in vitro* stimulation experiments, type II stimuli such as IL-4 and IL-13, inducing high arginase activity but no NO secretion, increased the expression levels of both FIZZ1 and Ym, whereas a combination of type I/type II stimuli (LPS plus IL-4), that augmented both arginase activity and NO secretion, enhanced the expression of Ym but not FIZZ1.

*In vitro* stimulation of macrophages from C57Bl/6 mice, but not from BALB/c mice, with IL-10 increased arginase activity, but did not induce detectable levels of FIZZ1 and Ym expression. Yet, the arginase activity induced by IL-10 was lower than the arginase activities induced by IL-4 or IL-13. Thus, it might be speculated that macrophages treated with IL-10 do not express FIZZ1 and Ym because they are not enough polarized towards an alternatively activated phenotype. Alternatively, it is possible that IL-10 induces a population of aaMϕ different from those expressing FIZZ1 and/or Ym.

Finally, we demonstrated that the differential expression of FIZZ1 and Ym can also be detected in total PEC, without isolating the macrophages via adherence. This can have important implications for situations where the amount of cells is too little to allow efficient purification. In addition, these results exclude the possibility that the observed differences in FIZZ1 and Ym expression are due to the induction of these genes during adherence.

In conclusion, the results of this study are in agreement with other studies showing a direct correlation between a type II cytokine environment and high expression of FIZZ1 and/or Ym (Falcone *et al.*, 2001; Chang *et al.*, 2001a; Webb *et al.*, 2001; and Nair *et al.*, 2003). We demonstrated that FIZZ1 and Ym constitute useful markers for the identification of both *in vitro*-elicited aaMϕ and aaMϕ derived from different organs in different infection models. This highlights that PLC$^{-/-}$ *T. b. brucei* infection, originally used for the molecular characterization of aaMϕ, leading to the identification of FIZZ1 and Ym as markers for alternative macrophage activation, is an appropriate *in vivo* model for the identification of genes that are differentially expressed in aaMϕ versus caMϕ. Such identification is a critical step in addressing the contribution and effector mechanisms of aaMϕ in the various biological processes with which they are associated, including immune suppression and immune deviation during parasitic infection (Loke *et al.*, 2000a,b), cancer (Mills *et al.*, 1992; Chang *et al.*, 2001b), pulmonary inflammation (Guo *et al.*, 2000) and wound healing (Gratchev *et al.*, 2001).

### MATERIALS AND METHODS

### Parasites and Animals

Female 8–12 weeks-old F1 (C57Bl/6 × BALB/c), BALB/c and C57Bl/6 mice (Harlan, Zeist, The Netherlands) were inoculated intraperitoneally with $5 \times 10^3$ phospholipase C-deficient mutant ($PLC^{-/-}$) *T. b. brucei* (Webb *et al.*, 1997) or Tc13 *T. congolense* (kindly provided by Dr Henry Tabel, University of Saskatchewan, Canada).

### Preparation of Macrophage Populations

Peritoneal exudate cells (PEC) from $PLC^{-/-}$ *T. b. brucei*-infected mice were collected in the early (6 days post-infection) or chronic (4–5 months post-infection) stage of infection. Spleen cells (SPC) from *T. congolense*-infected animals were harvested in the early (6 days post-infection) or late (8 weeks post-infection) phase of infection. The infections were timed so that at the time of PEC/SPC harvest, the mice at different stages of infection were age-matched. All cell cultures were performed in RPMI 1640 medium supplemented with 10% heat-inactivated fetal calf serum, $5 \times 10^{-5}$ M 2-mercapto-ethanol, 2 mM L-glutamine, 100 U/ml penicillin, 100 μg/ml streptomycin and 0.1 mm non-essential amino acids (all from GIBCO BRL). To obtain adherent cells, $2 \times 10^7$ PEC or $10^8$ SPC were dispensed in a 10-cm tissue culture dish (Falcon) and incubated at 37°C for 2–3 h in a humidified incubator containing 5% $CO_2$. Non-adherent cells and contaminating parasites were then washed away with RPMI 1640 pre-warmed at 37°C.

For *in vitro* stimulation, the adherent fraction of PEC from C57Bl/6 or BALB/c mice injected intraperitoneally with 3 ml of thioglycollate broth (BioMérieux, Marcy l'Etoile, France) 4 days prior to collection was cultured in the presence of the indicated stimuli for 48 h at the following final concentrations: 5 ng/ml IL-13 (R&D Systems), 100 U/ml IL-4 (Pharmingen), 100 U/ml IFN-γ (Pharmingen), 100 U/ml IL-10 (Pharmingen) and 100 ng/ml LPS (from *E. coli* Serotype 055:B5, Sigma).

### Quantification of Cytokines

Cytokines were quantified in cell culture supernatants using specific sandwich ELISAs for IFN-γ, IL-4 (PharMingen) or IL-13 (R&D Systems). ELISAs were performed as recommended by the suppliers. Reported cytokine levels are the maximal levels, i.e. observed after 1 day of culture for IL-4 and IL-13, and 3 days of culture for IFN-γ.

### Quantification of NO and Arginase Activity

Levels of NO in adherent cell culture supernatants were determined by quantifying $NO_2$ using Greiss reagent as described (Namangala *et al.*, 2001). Arginase activity was measured as previously reported (Namangala *et al.*, 2001). Briefly, $10^6$ cells were lyzed with 100 μl of 0.1% Triton X-100. After 30 min shaking at room temperature, arginase was activated in the presence of 100 μl 25 mM Tris–HCl pH 7.5 and 35 μl 10 mm $MnCl_2$ (10 min, 56°C). L-Arginine hydrolysis was conducted by incubating the cell lysate with 100 μl of 0.5 M L-arginine (pH 9.7) at 37°C for 1 h. The reaction was stopped by addition of 800 μl $H_2SO_4$ (96%)/$H_3PO_4$ (85%)/$H_2O$ (1v/3v/7v). The produced urea was quantified at 540 nm after addition of 40 μl of α-isonitrosopropiophenone (dissolved in 100% ethanol) followed by heating at 100°C for 20 min. One unit of enzyme was defined as the amount that catalyses the formation of 1 μmol of urea per min.

### General Molecular Techniques

Unless otherwise noted, nucleic acids were handled according to standard protocols (Sambrook *et al.*, 1989). Total RNA was prepared using Trizol reagent (GIBCO BRL).

### Semi-quantitative Reverse Transcriptase (RT)-PCR

One microgram of DNase-treated total RNA was reverse-transcribed using oligo(dT) and Superscript II reverse transcriptase (GIBCO BRL) following the manufacturers' recommendations. Each PCR cycle consisted of 1 min denaturation at 94°C, 45 s annealing at 54°C, and 1 min extension at 72°C. The PCR primers were FIZZ1 sense (5′-TCCCAGTGAATACTGATGAGA-3′), FIZZ1 antisense (5′-CCACTCTGGATCTCCCAAGA-3′), Ym sense (5′-GGGCATACCTTTATCCTGAG-3′), Ym antisense (5′-CCACTGAAGTCATCCATGTC-3′), β-actin sense (5′-ACACTGTGCCCATCTACGAG-3′), β-actin antisense (5′-TCAACGTCACACTTCATGATG-3′), ribosomal protein S12 sense (5′-GGAAGGCATAGCTGCTGGAG-GTGT-3′), and ribosomal protein S12 antisense (5-CCTCGATGACATCCTTGGCCTGAG-3′). The amplicon sizes were 213, 304, 381 and 367 bp for FIZZ1, Ym, β-actin and ribosomal protein S12, respectively. The Ym primers described above amplify both Ym1 and Ym2 sequences. The amount of template cDNA and the number of PCR cycles were optimized so that the analysis of PCR products could be carried out within the linear phase of amplification. β-actin or ribosomal protein S12 was used as control to ensure that the observed differences in the expression levels of each gene in different cells were not due to differences in the amount of template cDNA. The results of the PCR analyses were confirmed in at least three independent experiments.

***Acknowledgements***

The authors acknowledge the excellent technical assistance of Mrs E. Omasta, Miss M. Gobert and Mr. E. Vercauteren. This work was performed in the frame of an Interuniversity

Attraction Pole Program and was supported in part by a post-doctoral fellowship of the "Institute for Promotion of Innovation by Science and Technology in Flanders" (IWT-Vlaanderen, Brussels) to G. Raes., by the Fund for Scientific Research Flanders Fonds voor Wetenschappelijk Onderzoek Vlaanderen (FWO) by the UNDP/World Bank/WHO special programme for Research and Training in Tropical diseases (TDR), and by Vlaamse gewest (GBOU-IWT grant).

### References

Becker, S. and Daniel, E.G. (1990) "Antagonistic and additive effects of IL-4 and IFN-γ on human monocytes and macrophages: effects on Fc receptors, HLA-D antigens, and superoxide production", *Cell. Immunol.* **129**, 351–362.

Black, S.J. and Seed, R.J. (2001) *World Class Parasites: Volume 1 The African Trypanosomes* (Kluwer Academic Publishers, Dordrecht).

Buechler, C., Ritter, M., Orso, E., Langmann, T., Klucken, J. and Schmitz, G. (2000) "Regulation of scavenger receptor CD163 expression in human monocytes and macrophages by pro- and anti-inflammatory stimuli", *J. Leukoc. Biol.* **67**, 97–103.

Chang, N.-C.A., Hung, S.-I., Hwa, K.-Y., Kato, I., Chen, J.-E., Liu, C.-H. and Chang, A.C. (2001a) "A macrophage protein. Ym1, transiently expressed during inflammation is a novel mammalian lectin", *J. Biol. Chem.* **276**, 17497–17506.

Chang, C.I., Liao, J.C. and Kuo, L. (2001b) "Macrophage arginase promotes tumor cell growth and suppresses nitric oxide-mediated tumor cytotoxicity", *Cancer Res.* **61**, 1100–1106.

Conrad, D.J., Kuhn, H., Mulkins, M., Highland, E. and Sigal, E. (1992) "Specific inflammatory cytokines regulate the expression of human monocyte 15-lipoxygenase", *Proc. Natl Acad. Sci. USA* **89**, 217–221.

de Baetselier, P., Namangala, B., Noël, W., Brys, L., Pays, E. and Beschin, A. (2001) "Alternative versus classical macrophage activation during experimental African trypanosomosis", *Int. J. Parasitol.* **31**, 575–587.

Erwig, L.-P., Kluth, D.C., Walsh, G.M. and Rees, A.J. (1998) "Initial cytokine exposure determines function of macrophages and renders them unresponsive to other cytokines", *J. Immunol.* **161**, 1983–1988.

Falcone, F.H., Loke, P., Zang, X., MacDonal, A.S., Maizels, R.M. and Allen, J.E. (2001) "A *Brugia malayi* homolog of macrophage migration inhibitory factor reveals an important link between macrophages and eosinophil recruitment during nematode infection", *J. Immunol.* **167**, 5348–5354.

Gazzinelli, R.T., Wysocka, M., Hieny, S., Scharton-Kersten, T., Cheever, A., Kuhn, R., Muller, W., Trinchieri, G. and Sher, A. (1996) "In the absence of endogenous IL-10, mice acutely infected with *Toxoplasma gondii* succumb to a lethal immune response dependent on $CD4^+$ T cells and accompanied by overproduction of IL-12, IFN-γ and TNF-α", *J. Immunol.* **157**, 798–805.

Goerdt, S. and Orfanos, C.E. (1999) "Other functions, other genes: alternative activation of antigen-presenting cells", *Immunity* **10**, 137–142.

Goerdt, S., Bhardwaj, R. and Sorg, C. (1993) "Inducible expression of MS-1 high molecular weight protein by endothelial cells of continuous origin and by dendritic cells/macrophages *in vivo* and *in vitro*", *Am. J. Pathol.* **142**, 1409–1422.

Gratchev, A., Guilot, P., Hakiy, N., Politz, O., Orfanos, C.E., Schledzewski, K. and Goerdt, S. (2001) "Alternatively activated macrophages differentially express fibronectin and its splice variants and the extracellular matrix protein βIG-H3", *Scand. J. Immunol.* **53**, 386–392.

Guo, L., Johnson, R.S. and Schuh, J.C.L. (2000) "Biochemical characterization of endogenously formed eosinophilic crystals in the lungs of mice", *J. Biol. Chem.* **275**, 8032–8037.

Henson, P.M. and Riches, D.W. (1994) "Modulation of macrophage maturation by cytokines and lipid mediaters: a potential role in resolution of pulmonary inflammation", *Ann. N.Y. Acad. Sci.* **725**, 298–308.

Holcomb, I.N., Kabakoff, R.C., Chan, B., Baker, T.W., Gurney, A., Henzel, W., Nelson, C., Lowman, H.B., Wright, B.D., Skelton, N.J., Frantz, G.D., Tumas, D.B., Peale, F.V., Shelton, D.L. and Hebert, C.C. (2000) "FIZZ1, a novel cysteine-rich secreted protein associated with pulmonary inflammation, defines a new gene family", *EMBOQ. J.* **19**, 4046–4055.

Hunter, C.A., Ellis-Neyes, L.A., Slifer, T., Kanaly, S., Grunig, G., Fort, M., Rennick, D. and Araujo, F.G. (1997) "IL-10 is required to prevent immune hyperactivity during infection with *Trypanosoma cruzi*", *J. Immunol.* **158**, 3311–3316.

Janeway, C.A., Jr. and Travers, P. (1996) *Immunobiology: The Immune System in Health and Disease* (Current Biology Ltd., London).

Kodelja, V., Muller, C., Tenorio, S., Schebesch, C., Orfanos, C.E. and Goerdt, S. (1997) "Differences in angiogenic potential of classically versus alternatively activated macrophages", *Immunobiology* **197**, 478–493.

Kodelja, V., Müller, C., Politz, O., Hakij, N., Orfanos, C.E. and Goerdt, S. (1998) "Alternative macrophage activation-associated CC-chemokine-1α, a novel structural homologue of macrophage inflammatory protein-1 with a Th2-associated expression pattern", *J. Immunol.* **160**, 1411–1418.

Li, C., Corraliza, I. and Langhorne, J. (1999) "A defect in interleukin-10 leads to enhanced malarial disease in *Plasmodium chabaudi* infection in mice", *Infect. Immun.* **67**, 4435–4442.

Loke, P., MacDonald, A.S. and Allen, J.E. (2000a) "Antigen-presenting cells recruited by *Brugia malayi* induce Th2 differentiation of naïve $CD4^+$ T cells", *Eur. J. Immunol.* **30**, 1127–1135.

Loke, P., MacDonald, A.S., Robb, A., Maizels, R.M. and Allen, J.E. (2000b) "Alternatively activated macrophages induced by nematode infection inhibit proliferation via cell-to-cell contact", *Eur. J. Immunol.* **30**, 2669–2678.

Mills, C.D., Shearer, J., Evans, R. and Caldwell, M.D. (1992) "Macrophage arginine metabolism and the inhibition or stimulation of cancer", *J. Immunol.* **149**, 2709–2714.

Munder, M., Eichmann, K. and Modolell, M. (1998) "Alternative metabolic states in murine macrophages reflected by the nitric oxide synthase/arginase balance: competitive regulation by $CD4^+$ T cells correlates with Th1/Th2 phenotype", *J. Immunol.* **160**, 5347–5354.

Nair, M.G., Cochrane, D.W. and Allen, J.E. (2003) "Macrophages in chronic type 2 inflammation have a novel phenotype characterized by the abundant expression of Yml and FIZZ1 that can be partly replicated *in vitro*", *Immunol. Letters* **85**, 173–180.

Nöel, W., Hassanzadeh, Gh.G., Raes, G., Namangala, B., Daems, I., Brys, L., Brombacher, F., de Baetselier, P. and Beschin, A. (2002) "Infection stage-dependent modulation of macrophage activation in *Trypanosoma congolense*-resistant and -susceptible mice", *Infect. Immun.* **70**, 6180–6187.

Namangala, B., de Baetselier, P., Brijs, L., Stijlemans, B., Noël, W., Pays, E., Carrington, M. and Beschin, A. (2000) "Attenuation of *Trypanosoma brucei* is associated with reduced immunosuppression and concomitant production of Th2 lymphokines", *J. Infect. Dis.* **181**, 1110–1120.

Namangala, B., de Baetselier, P., Noël, W., Brys, L. and Beschin, A. (2001) "Alternative versus classical macrophage activation during experimental African trypanosomosis", *J. Leukoc. Biol.* **69**, 387–396.

Raes, G., de Baetselier, P., Noël, W., Beschin, A., Brombacher, F. and Hassanzadeh, Gh. G. (2002) "Differential expression of FIZZ1 and Ym1 in alternatively versus classically activated macrophages", *J. Leukoc. Biol.* **71**, 597–602.

Sambrook, J., Fritsch, E. and Maniatis, T. (1989) *Molecular Cloning: A Laboratory Manual*, 2nd Ed. (Cold Spring Harbor Laboratory, New York).

Schebesch, C., Kodelja, V., Muller, C., Hakij, N., Bisson, S., Orfanos, C.E. and Goerdt, S. (1997) "Alternatively activated macrophages actively inhibit proliferation of peripheral blood lymphocytes and $CD4^+$ T cells *in vitro*", *Immunology* **92**, 478–486.

Stein, M., Keshav, S., Harris, N. and Gordon, S. (1992) "Interleukin 4 potently enhances murine macrophage mannose receptor activity: a marker of alternative immunologic macrophage activation", *J. Exp. Med.* **176**, 287–292.

Steppan, C.M., Brown, E.J., Wright, C.M., Bhat, S., Banerjee, R.R., Dai, C.Y., Enders, G.H., Silberg, D.G., Wen, X., Wu, G.D. and Lazar, M.A. (2000) "A family of tissue-specific resistin-like molecules", *Proc. Natl Acad. Sci. USA* **98**, 502–506.

Sun, Y.-J., Chang, N.-C.A., Hung, S.-I., Chang, A.C., Chou, C.-C. and Hsiao, C.D. (2001) "The crystal structure of a novel mammalian lectin, Ym1, suggests a saccharide binding site", *J. Biol. Chem.* **276**, 17507–17514.

te Velde, A.A., Huijbens, R.J., de Vries, J.E. and Figdor, C.G. (1990) "IL-4 decreases Fcγ R-mediated cytotoxic activity of human monocytes", *J. Immunol.* **144**, 3046–3051.

Uzonna, J.E., Kaushik, R.S., Gordon, J.R. and Tabel, H. (1999) "Cytokines and antibody responses during *Trypanosoma congolense* infections in two inbred mouse strains that differ in resistance", *Parasite Immunol.* **21**, 57–71.

Webb, H., Carnall, N., Vanhamme, L., Rolin, S., van den Abbeele, J., Welburn, S., Pays, E. and Carrington, M. (1997) "The GPI-phospholipase C of *Trypanosoma brucei* is not essential but influences parasitemia in mice", *J. Cell Biol.* **139**, 103–114.

Webb, D.C., McKenzie, A.N.J. and Foster, P.S. (2001) "Expression of the Ym2 lectin-binding protein is dependent on interleukin (IL)-4 and IL-13 signal transduction", *J. Biol. Chem.* **276**, 41969–41976.

